# Frailty, Physical Function Impairment and Pulmonary Function in Aging Men with and without HIV from the Multicenter AIDS Cohort Study (MACS)

**DOI:** 10.21203/rs.3.rs-4908040/v1

**Published:** 2024-09-10

**Authors:** Mona Abdo, Ken M Kunisaki, Alison Morris, Valentina Stosor, Dong Chang, Gypsyamber D’Souza, Kristina Crothers, Madiha Abdel-Maksoud, Carolyn DiGuiseppi, Todd T Brown, Samantha MaWhinney, Kristine M Erlandson

**Affiliations:** Colorado School of Public Health; Minneapolis Veterans Affairs Health Care System; University of Pittsburgh School of Medicine; Northwestern University Feinberg School of Medicine; Harbor–UCLA Medical Center; Johns Hopkins Bloomberg School of Public Health; Veterans Affairs Puget Sound and University of Washington; Colorado School of Public Health; Colorado School of Public Health; Johns Hopkins School of Medicine; Colorado School of Public Health; University of Colorado-Anschutz Medical Campus

**Keywords:** Physical Function, Pulmonary Function, HIV, Aging

## Abstract

**Background:**

People aging with HIV (PAWH) experience greater impairment in physical and pulmonary function than individuals aging without HIV. We examined whether baseline physical function was associated with subsequent pulmonary impairments.

**Methods:**

Associations of frailty and physical function (gait speed [m/sec], grip strength [kg]) with pulmonary function (< 80% predicted diffusing capacity for carbon monoxide [DL_CO_] and forced expiratory volume [FEV_1_]) 3 years later were modeled; age, HIV status, and smoking were assessed as effect modifiers.

**Results:**

Among1,024 men, (54% PAWH, 10% frail, 51% pre-frail), mean (SD) age = 53 (12) years, cumulative smoking = 12 (19) pack-years, gait speed = 1.1 (0.2) m/sec, and grip strength = 36.6 (9.2) kg. Frailty, pre-frailty, and weak grip strength were associated with higher odds of subsequent impaired DL_CO_ and FEV_1_. Slow gait speed was associated with higher odds of DL_CO_ impairment but not FEV_1_. No statistically significant modifications were found.

**Conclusion:**

Interventions to improve physical function may help preserve pulmonary function.

## Introduction

The life expectancy for people aging with HIV (PAWH) has increased due to effective antiretroviral therapy (ART). In 2019, about half of U.S. adults with HIV were over the age of 50 [1–4]. PAWH are at higher risk of developing adverse health outcomes earlier with greater severity, including frailty and physical function impairments [5–7]. Frailty and pulmonary disease may co-occur due to shared risk factors and pathophysiological mechanisms [5–14]. Research suggests a bidirectional association between frailty and respiratory impairments [14]. We, and others, have shown pulmonary impairment is associated with increased subsequent frailty risk, decreased gait speed, and weaker grip strength, among men with and without HIV [14–18]. Here we hypothesize that frailty and physical function impairments (slow gait speed and weak grip strength) are associated with subsequent impaired pulmonary function, and that associations would be greater among PAWH, older adults, and smokers.

## Methods

### Study Population

The MACS, now part of the Multicenter AIDS Cohort Study/Women’s Interagency HIV Combined Cohort Study (MACS/WIHS-CCS) [19], began in 1984 and enrolled men with or at risk of HIV at four study centers (Baltimore/Washington DC, Chicago, Pittsburgh/Columbus, and Los Angeles) and three enrollment cohorts (before 1995, between 2001–2009, and 2010 and later). Institutional review board approval was obtained at each site. MACS details have been published elsewhere; in brief, participants completed semi-annual visits for demographic, clinical, and laboratory assessments [20, 21]. Pulmonary function tests (PFTs) were conducted from 2017–2019. Participants were invited to undergo PFTs; those who consented, completed PFTs and had measurements that passed quality control standards were included here [22, 23]. The visit three years prior to PFTs was considered baseline for frailty, gait, and grip assessments. If the baseline visit was missing frailty or physical function assessments, then the next visit (up to one year prior to PFTs) was used. Participants who seroconverted during follow-up were excluded (n = 13). Detailed information about PFT testing and exposure measures (frailty, gait speed, grip strength) are included in supplemental material.

### Effect Modifiers

Age, HIV serostatus, and cumulative pack-years of smoking (at baseline) were examined as potential effect modifiers on the associations of frailty and physical function with pulmonary function.

### Covariates

Baseline HIV serostatus, enrollment center, race (self-reported and categorized into: Black, White, Other), age, cumulative pack-years of smoking and enrollment cohort were included in all models. In models examining interactions of age with DL_CO_ or FEV_1_ (using absolute measurement), height (in cm) was also included. Potential baseline confounders included: education level, weight, body mass index (BMI), cardiovascular risk (Framingham coronary heart disease 10-year risk score %), diabetes defined as hemoglobin A1c ≥ 6.5% or fasting glucose ≥ 126 mg/dL or self-reported diagnosis of diabetes with medication use, use of cholesterol lowering medication, treatment for depression, current or prior confirmed diagnosis of kidney disease, hepatitis C seropositivity, hepatitis B (surface antigen positive or resolved vs negative), and any use since last visit of alcohol, marijuana, cocaine and/or heroin.

### Statistical Analysis

Statistical analyses utilized SAS v9.4 (Cary, NC). Logistic regression models determined associations of frailty and physical function with pulmonary function. Interaction terms between each exposure (frailty, gait speed, and grip strength) and age, HIV serostatus, or smoking were explored to determine if they separately modified associations between frailty or physical function and pulmonary function **(supplemental materials).** Exploratory analyses examined if frailty components other than gait speed or grip strength (i.e., weight loss, exhaustion, or low physical activity) were associated with pulmonary function impairment.

### Minimal Clinically Important Differences (MCID) for Effect Modification

Interaction terms between frailty or physical function with age, HIV serostatus, or smoking were considered separately. A minimal clinically important difference (MCID) was determined based on the literature and/or clinical expertise [23–26] (**supplemental materials)**.

## Results

Of 1,133 men with PFTs, 1,024 and 1,007 met inclusion criteria for DL_CO_ and FEV_1_ analyses, respectively. Overall, 54% and 55% of the DL_CO_ and FEV_1_ participants, respectively, were PAWH. Cohort characteristics are reported in Table 1. Sixteen and 14 participants with DL_CO_ and FEV_1_ measures, respectively were missing frailty measurement. On average, participants had 5.7 visits (SD = 1.7) between frailty and PFT measurements.

### Adjusted Associations between Frailty or Physical Function and Pulmonary Function

**Figure 2:**
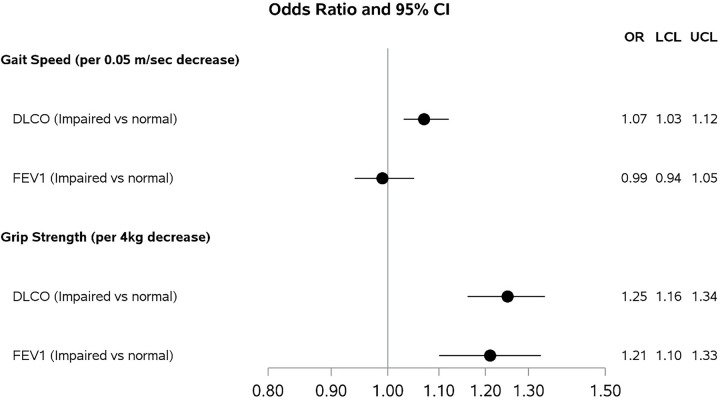
Odds Ratios and 95% Confidence Intervals from Adjusted Logistic Regression Models for the Associations of Gait Speed (m/sec) and Grip Strength (kg) with subsequent impairment in DLco* and FEV_1_**. Gait Speed Models: *DL_CO_ models adjusted for Age, Cohort of enrollment, Center of enrollment, Race, HIV serostatus, and Cumulative pack-years of smoking and Framingham hard coronary 10-year risk score. ** FEV_1_ models adjusted for Age, Cohort of enrollment, Center of enrollment, Race, HIV serostatus, and Cumulative pack-years of smoking, Framingham hard coronary 10-year risk score, education, diabetes status, high cholesterol, treatment for depression, and cocaine use. Grip Strength Models: *DL_CO_ models adjusted for Age, Cohort of enrollment, Center of enrollment, Race, HIV serostatus, and Cumulative pack-years of smoking. ** FEV_1_ models adjusted for Age, Cohort of enrollment, Center of enrollment, Race, HIV serostatus, and Cumulative pack-years of smoking and Framingham hard coronary 10-year risk score

**Figure 3:**
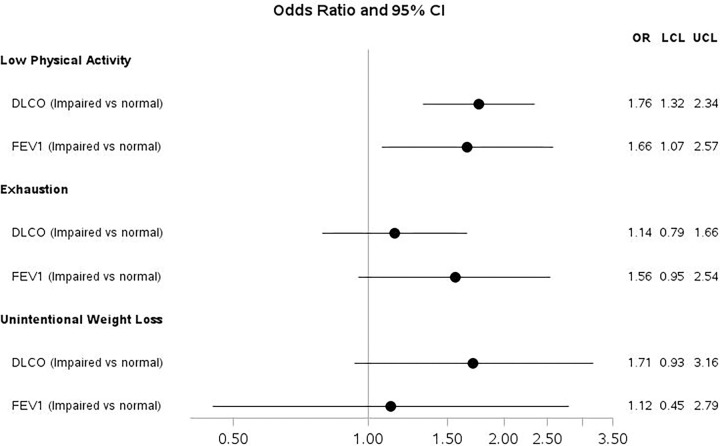
Odds Ratios and 95% Confidence Intervals from Adjusted Logistic Regression Models for the Associations of Gait Speed (m/sec) and Grip Strength (kg) with subsequent impairment in DLco* and FEV_1_**. *DL_CO_ models adjusted for Age, Cohort of enrollment, Center of enrollment, Race, HIV serostatus, and Cumulative pack-years of smoking ** FEV_1_ models adjusted for Age, Cohort of enrollment, Center of enrollment, Race, HIV serostatus, and Cumulative pack-years of smoking, diabetes, and Framingham hard coronary 10-year risk score

Compared to non-frail participants, the odds of impaired DL_CO_ (< 80% predicted) were 1.94 [(95% CI 1.42, 2.65); p < 0.001] times higher for pre-frail participants and 2.53 [(1.54, 4.15); p < 0.001] times higher for frail participants, (Fig. 2, **Supplemental Table 1a**). Similarly, compared to non-frail participants, the odds of impaired FEV_1_ were 1.44 times higher than pre-frail participants and 2.32 times higher for frail participants compared to non-frail ([95% CI 0.90, 2.32]; p = 0.13; [95% CI 1.24, 4.32]; p = 0.008, respectively, Fig. 2, **Supplemental Table 1b**). Every 0.05 m/sec decrease in gait speed was associated with greater odds of DL_CO_ impairment (OR = 1.07 [95% CI 1.03, 1.12]; p < 0.001, Fig. 3, **Supplemental Table 2a**) but not with FEV_1_ impairment (OR = 0.99 [95% CI 0.94, 1.05]; p = 0.82, Fig. 3, **Supplemental Table 2b**). Every 4 kg decrease in grip strength was associated with greater odds of both DL_CO_ (OR = 1.25 [95% CI 1.16, 1.34]; p < 0.001, Fig. 3, **Supplemental Table 3a**) and FEV_1_ impairment (OR = 1.21 [95% CI 1.10, 1.33]; p < 0.001, Fig. 3, **Supplemental Table 3b**).

### Effect Modification

Inconclusive results were found when exploring age, HIV status, or smoking modification of frailty on DL_CO_ and FEV_1_ (**Supplemental Tables 4–11**) and age modification of grip strength on FEV_1_ (**Supplemental Table 12**). Therefore, primary results were presented without these interactions.

Neither age, HIV, nor smoking modified associations between gait speed or grip strength and DL_CO_ (**Supplemental Tables 13–18**) or between gait speed and FEV_1_ (**Supplemental Tables 19–21**). Neither HIV nor smoking modified the association between grip strength and FEV_1_ (**Supplemental Tables 22–23**-**20).**

### Exploratory Analysis

In exploratory analyses, we examined associations of other frailty components (unintentional weight loss, exhaustion, and low physical activity) with subsequent impaired pulmonary function. Participants reporting low physical activity had higher odds of DL_CO_ and FEV_1_ impairment compared to participants not reporting low physical activity (OR = 1.76 [95% CI 1.32, 2.34]; p < 0.01, OR = 1.66 [95% CI 1.07, 2.57]; p = 0.02, respectively, Fig. 3). Unintentional weight loss and exhaustion were not significantly associated with subsequent pulmonary function (Fig. 3).

## Discussion

We examined the associations between frailty and frailty components with subsequent pulmonary function in a large study of men with and without HIV, across a range of pulmonary function. Frailty, decreased grip strength, and low physical activity were significantly associated with increased odds of having impaired DL_CO_ and FEV_1_, and slow gait speed was associated with impaired DL_CO_. Our results expand on the literature by showing associations between frailty and subsequent pulmonary function across multiple measures. Surprisingly, associations between frailty, gait speed, and grip strength with DL_CO_ and FEV_1_ were generally similar regardless of age, HIV serostatus, and smoking history. Considering this and our previous study [18], we propose a bidirectional association between pulmonary function and physical function impairment or frailty, as has been suggested in the general population [14]. Interventions that target components of both COPD and frailty may be the most effective in preventing decline in both conditions.

We did not find differences in associations between physical function or frailty and pulmonary function by HIV serostatus or age. We did observe a higher non-significant percentage of PAWH with impaired DL_CO_ compared to men without HIV (36% vs 31%, chi-square p-value = 0.09), and PAWH also had a slightly higher non-significant percentage of frailty (10.3% vs 9.4%, chi-square p-value = 0.32). The lack of modification by HIV serostatus could result from the majority of the PAWH included in this analysis having limited disease progression with 86% having viral load ≤ 200 copies/ml and 74% having a CD4 + count over 500. Lack of modification by age could be due to one-time pulmonary function testing or the relatively narrow age range.

This study has several strengths. It is a multicenter study with a large sample size including men with and without HIV. We used well-validated objective physical function measures of gait speed and grip strength and lung function. The extensive demographic and health-related data allowed us to adjust for numerous covariates. This study also has limitations. Although the MACS has more recently combined with the Women’s Interagency HIV Study (comparable data not yet available in WIHS), this study only included U.S. men, > 90% of whom were White or Black, and study results may not be generalizable to women, other racial/ethnic groups, or non-U.S. populations. We only looked at single measurements for both physical function/frailty and pulmonary function (at different time points); so, could not examine changes over time. Participants with impaired pulmonary function at the 3-year follow-up may have had baseline impairments as well. Future studies are needed to determine temporality and confirm bidirectionality.

In conclusion, among men with and without HIV, we found a strong association between frailty and impaired physical function with subsequent pulmonary function impairment. Combined with our prior analyses, these fundings support a bidirectional association between pulmonary function with physical function and frailty [18]. A better understanding of mechanistic pathways underlying both frailty and pulmonary functions are needed to develop improved treatments to reduce declines in physical and pulmonary function and improve overall quality of life.

## Figures and Tables

**Figure 1:**
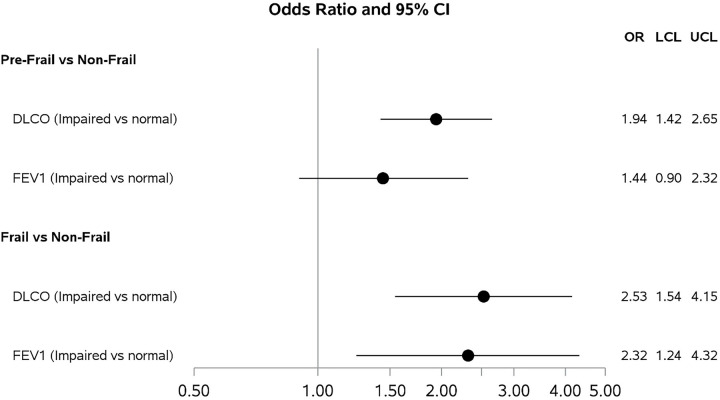
Odds Ratios and 95% Confidence Intervals from Adjusted Logistic Regression Models for the Association of Frailty with subsequent impairment in DLco* and FEV_1_**. *DLCO models adjusted for Age, Cohort of enrollment, Center of enrollment, Race, HIV serostatus, and Cumulative pack-years of smoking ** FEV1 models adjusted for Age, Cohort of enrollment, Center of enrollment, Race, HIV serostatus, and Cumulative pack-years of smoking and Framingham hard coronary 10-year risk score

**Table 1:** Baseline Characteristics of the Cohort (DLCO and FEV1).

Variable	DLCO Cohort Overall (n = 1,024)	FEV1 Cohort Overall (n = 1,007)

**Age (years) mean (SD)**	52.8 (12.0)	52.8 (12.1)

**Race** White Black Other	656 (64%)	640 (64%)
	278 (27%)	273 (27%)
	90 (9%)	94 (9%)

**Center of Enrollment**	208 (20%)	206 (20%)
Baltimore/Washington DC	257 (25%)	243 (24%)
Chicago	312 (31%)	311 (31%)
Pittsburgh/Columbus	247 (24%)	247 (25%)
Los Angeles		

**Cohort of Enrollment**	469 (46%)	454 (45%)
Before 1995	400 (39%)	397 (39%)
2001–2010	155 (15%)	156 (16%)
2010+		

**Education level**	216 (21%)	219 (22%)
12th grade or less	275 (27%)	271 (27%)
At least one year of college but no degree Four-year college degree or more	533 (52%)	517 (51%)

**HIV Serostatus**	552 (54%)	552 (55%)
HIV + HIV−	472(46%)	455 (45%)

**CD4 Count cells/mm^3^ (for HIV+) mean (SD)**	692.1 (294.0)	698.7 (303.7)

**Viral Load ≤ 200 copies/ml (for HIV+)**	476 (86%)	478 (87%)

**Smoking Status** Never Smoker Former Smoker	328 (32%)	321 (32%)
Current Smoker	440 (43%)	438 (44%)
	254 (25%)	246 (24%)

**Cumulative Pack Years of Smoking mean (SD)**	12.0 (18.7)	12.1 (19.1)

**Weight (kg) mean (SD)**	84.4 (17.1)	84.2 (17.0)

**Body Mass Index (BMI, kg/m^2^) mean (SD)**	26.9 (4.9)	26.9 (5.0)

**Alcohol Use**	187 (18%)	191 (19%)
None	495 (49%)	488 (49%)
1–3 drinks/week	248 (24%)	235 (23%)
4–13 drinks/week	87 (9%)	87 (9%)
More than 13 drinks/week		

**Framingham Coronary Heart Disease 10-Year Risk (%) mean (SD)**	8.0 (7.3)	8.1 (7.3)

**Framingham Hard Coronary Heart Disease 10-Year Risk (%) mean (SD)**	8.6 (6.0)	8.6 (6.0)

**Diabetes Status**	136 (14%)	139 (15%)

**Treatment for Depression**	140 (14%)	139 (14%)

**On Cholesterol Lowering Medication**	350 (34%)	338 (34%)

**Heroin Use**	18 (2%)	17 (2%)

**Cocaine Use**	83 (8%)	83 (8%)

**Injection Drug Use**	18 (2%)	19 (2%)

**Hash/Marijuana Use**	324 (32%)	312 (31%)

**Kidney Disease (current or prior)**	21 (2%)	25 (3%)

**Hepatitis B (active or resolved)**	524 (52%)	504 (50%)

**Hepatitis C seropositivity**	54 (5%)	48 (5%)

**Gait Speed (m/sec) mean (SD)**	1.1 (0.2)	1.1 (0.2)

**Grip Strength (kg) mean (SD)**	36.6 (9.2)	36.3 (9.4)

**Frailty**	397 (39%)	385 (39%)
Non-Frail	510 (51%)	491 (50%)
Pre-Frail	100 (10%)	114 (11%)
Frail		

**DLCO Impaired (< 80% predicted)**	342 (33%)	----

**FEV1 Impaired (< 80% predicted)**	---	128 (13%)

* DL_CO_ models adjusted for Age, Cohort of enrollment, Center of enrollment, Race, HIV serostatus, and Cumulative pack-years of smoking

** FEV_1_ models adjusted for Age, Cohort of enrollment, Center of enrollment, Race, HIV serostatus, and Cumulative pack-years of smoking and Framingham hard coronary 10-year risk score

Gait Speed Models:

* DL_CO_ models adjusted for Age, Cohort of enrollment, Center of enrollment, Race, HIV serostatus, and Cumulative pack-years of smoking and Framingham hard coronary 10-year risk score.

** FEV_1_ models adjusted for Age, Cohort of enrollment, Center of enrollment, Race, HIV serostatus, and Cumulative pack-years of smoking, Framingham hard coronary 10-year risk score, education, diabetes status, high cholesterol, treatment for depression, and cocaine use.

Grip Strength Models:

* DL_CO_ models adjusted for Age, Cohort of enrollment, Center of enrollment, Race, HIV serostatus, and Cumulative pack-years of smoking.

** FEV_1_ models adjusted for Age, Cohort of enrollment, Center of enrollment, Race, HIV serostatus, and Cumulative pack-years of smoking and Framingham hard coronary 10-year risk score

* DL_CO_ models adjusted for Age, Cohort of enrollment, Center of enrollment, Race, HIV serostatus, and Cumulative pack-years of smoking

** FEV_1_ models adjusted for Age, Cohort of enrollment, Center of enrollment, Race, HIV serostatus, and Cumulative pack-years of smoking, diabetes, and Framingham hard coronary 10-year risk score

## Data Availability

Data in this manuscript were collected by MACS/WIHS Combined Cohort Study (MWCCS). The data are not publicly available and can be requested through the MWCCS.
